# Thermal Effects during Bone Preparation and Insertion of Osseointegrated Transfemoral Implants

**DOI:** 10.3390/s21186267

**Published:** 2021-09-18

**Authors:** Emir Benca, Beatrice Ferrante, Martin Zalaudek, Lena Hirtler, Alexander Synek, Franz M. Kainberger, Reinhard Windhager, Rickard Brånemark, Gerhard M. Hobusch, Ewald Unger

**Affiliations:** 1 Department of Orthopedics and Trauma Surgery, Medical University of Vienna, 1090 Vienna, Austria; beatrice.ferrante96@gmail.com (B.F.); reinhard.windhager@meduniwien.ac.at (R.W.); gerhard.hobusch@meduniwien.ac.at (G.M.H.); 2 Department of Chemistry, Materials and Chemical Engineering “Giulio Natta”, Politecnico di Milano, 20133 Milano, Italy; 3 Department of Biomedical Imaging and Image-Guided Therapy, Medical University of Vienna, 1090 Vienna, Austria; martin.zalaudek@meduniwien.ac.at (M.Z.); franz.kainberger@meduniwien.ac.at (F.M.K.); 4 Centre for Anatomy and Cell Biology, Division of Anatomy, Medical University of Vienna, 1090 Vienna, Austria; lena.hirtler@meduniwien.ac.at; 5 Institute of Lightweight Design and Structural Biomechanics, TU Wien, 1060 Vienna, Austria; asynek@ilsb.tuwien.ac.at; 6 Department of Orthopaedics, Gothenburg University, 40530 Gothenburg, Sweden; rickard.branemark@orthop.gu.se; 7 Biomechatronics Group, Massachusetts Institute of Technology, Cambridge, MA 02139, USA; 8 Center for Medical Physics and Biomedical Engineering, Medical University of Vienna, 1090 Vienna, Austria; ewald.unger@meduniwien.ac.at

**Keywords:** drilling, femur, heat, osseointegrated implant, speed

## Abstract

Background: The preparation of bone for the insertion of an osseointegrated transfemoral implant and the insertion process are performed at very low speeds in order to avoid thermal damages to bone tissue which may potentially jeopardize implant stability. The aim of this study was to quantify the temperature increase in the femur at different sites and insertion depths, relative to the final implant position during the stepwise implantation procedure. Methods: The procedure for installation of the osseointegrated implant was performed on 24 femoral specimens. In one specimen of each pair, the surgery was performed at the clinically practiced speed, while the speed was doubled in the contralateral specimen. Six 0.075 mm K fine gauge thermocouples (RS Components, Sorby, UK) were inserted into the specimen at a distance of 0.5 mm from the final implant surface, and six were inserted at a distance of 1.0 mm. Results: Drilling caused a temperature increase of <2.5 °C and was not statistically significantly different for most drill sizes (0.002 < *p* < 0.845). The mean increase in temperature during thread tapping and implant insertion was <5.0 °C, whereas the speed had an effect on the temperature increase during thread tapping. Conclusions: Drilling is the most time-consuming part of the surgery. Doubling the clinically practiced speed did not generate more heat during this step, suggesting the speed and thus the time- and cost-effectiveness of the procedure could be increased. The frequent withdrawal of the instruments and removal of the bone chips is beneficial to prevent temperature peaks, especially during thread tapping.

## 1. Introduction

The traditional method of attaching prostheses for patients who have undergone a transfemoral amputation is by means of socket prostheses. To achieve optimal prosthetic mobility, an appropriate socket suspension is crucial. Socket-related problems, such as discomfort, perspiration, and skin problems, have been shown to jeopardize comfort and mobility in patients with lower-limb amputations [1]. For the past three decades, bone anchorage has been representing another alternative to conventional suspension techniques. The first installed implant was a threaded, cylinder-like, fully implanted component known as a “fixture” made of titanium providing the anchorage “by the formation of bony tissue around it without the growth of fibrous tissue at the bone-implant interface” in a bilateral transfemoral amputee in 1990 [2,3]. Today, this implant system has been further developed and is known as Osseointegrated Prostheses for the Rehabilitation of Amputees (OPRA) (Integrum AB, Mölndal, Sweden). The surgical technique for the OPRA system in transfemoral amputees requires surgical exposure of the femur, opening of the intramedullary canal, drilling, thread tapping, and fixture insertion. During drilling, a significant amount of diaphyseal cortical bone is removed. Drilling is performed in 0.5 mm increments, from 10.5 to 24.0 mm drills, depending on the size of the chosen fixture, to a depth of 100 mm and is followed by thread tapping. The smallest fixtures with 16.0 or 16.5 mm diameter have a thread pitch of 1.25 mm, and the others have a thread pitch of 1.75 mm. Finally, the fixture, matching the diameter of the thread tap, is inserted.

High friction during drilling, thread tapping, and fixture insertion may lead to heat-induced bone tissue injury. Consequently, overheating could induce bone tissue necrosis, inhibit bone microcirculation, and activate bone marrow macrophages, thus jeopardizing the regenerative capacity of the bone. Furthermore, these heat-induced tissue injuries endanger primary healing and osseointegration. They result in reduced initial implant stability and thus lead to early implant failure [4]. Therefore, the surgeon is required to perform the aforementioned steps at a slow speed, significantly affecting the duration of the surgery. In the past, several studies have examined heat generation in bone during surgical preparation. It has been shown that bone tissue necrosis may occur if the critical temperature threshold of 47 °C is exceeded for more than 1 min and that heat stress at 50 °C for 1 min or 47 °C for 5 min hinders osteoblast regeneration and causes bone resorption and conversion to adipocytes, thus failing to form osseous tissue [5]. Heat generation was primarily studied in dental surgery, and to the best knowledge of the authors, no study exists on heat generation during bone preparation and insertion of osseointegrated transfemoral implants. The purpose of this study was to assess the change in temperature caused by different surgical steps at a very close distance to the implant position for different speeds. Furthermore, the temperature increase was correlated with the bone’s morphological data. The aim is to minimize surgery duration without causing thermal damage to the bone tissue and possibly jeopardizing implant stability.

## 2. Materials and Methods

**Figure 1:**
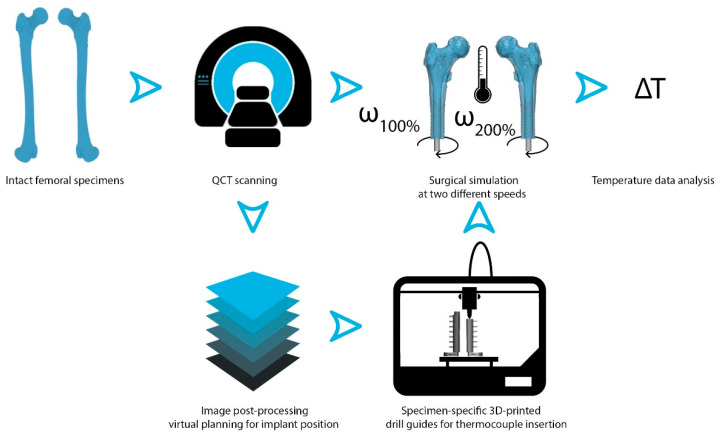
Graphical abstract of the project: Twenty-four paired femoral specimens were scanned using a QCT scanner. Images were postprocessed to create specimen-specific drill guides to facilitate the placement of thermocouples in each specimen. The surgery was performed at the clinically practiced speed (ω_100%_) in one femur of each pair and at the doubled speed in the contralateral specimen (ω_200%)_. Consequently, data analysis was performed to assess if the higher speed generates more heat.

A graphical abstract of the project is given in Figure 1.

### 2.1. Specimens

A total of 24 paired specimens of human femora, obtained from donors to the Centre for Anatomy and Cell Biology, Medical University of Vienna, were used. The donors had given written consent for their bodies to be used for research and education. The study was approved by the Ethics Committee of the Medical University of Vienna (1876/2019). The mean age of the six female and six male donors was 79.5 ± 11.2 years (range: 64–100). In order to prevent any change in the mechanical properties of the bone tissue, the specimens were fresh frozen at −20 °C and exposed to room temperature 12 h before preparation. Femora were preselected based on the diameter of the bone and its medullar cavity to fit the 18.0 × 80 mm BioHelix fixture. Specimens were inspected for fracture, presence of lesions, and prosthetic restoration. According to the previously set criteria, all specimens proved valid for the study. Finally, the femora were amputated at a random shaft height. The amputation height was chosen arbitrarily to better reflect the clinical reality.

### 2.2. Radiological Imaging and Image Segmentation

Following preparation, all specimens underwent radiological assessment for bone mineral density (BMD) and morphology. The Horizon DXA system (Hologic, Inc., Marlborough, MA, USA) was used to assess the bone mineral density (g/cm^2^) of the proximal femur. QCT image sequences were acquired with the SOMATOM Force 128-slice dual-source CT scanner (Siemens Healthcare GmbH, Erlangen, Germany) with the following parameters for tube A and tube B: voltage: 120 and 150 kV, intensity: 270 and 540 mAs, respectively. QCT image sequences were reconstructed at a slice thickness of 0.6 mm and position increment of 0.4 mm using the Qr69 kernel and the ‘Bone’ window.

Images were segmented and postprocessed for preoperative planning and creation of drill guides (see Experimental Setup) using Mimics Research 21.0 and 3-Matic Research 13.0 (both Materialise NV, Leuven, Belgium). Cortical width (mm) was measured at 12 sites (6 on medial and 6 on lateral side) along the axis of the implant in the coronal plane.

### 2.3. Surgical Procedure

A single senior surgeon (GMH) specialized in tumor orthopedics and trained for the OPRA Implant System performed all surgical steps.

The surgical steps included drilling, thread tapping, withdrawal of the drills and the thread tap, and implant insertion and were performed successively without time interruptions. Drilling started with the largest drill diameter fitting the distal entry of the medullar cavity of the specific specimen ranging from 10.5 to 16.5 mm. All steps were performed consecutively without time interruption to mimic the clinical setting.

In the first group of specimens, the surgeon performed all surgical steps following recommended rotational speeds for drilling, thread tapping, instrument withdrawal, and implant insertion mimicking clinical situations (ω_100%_). This speed was assessed from a previously recorded real surgery using a video camera. The rotational speed was similar for all surgical steps and corresponded to approximately ω_100%_ = 60°/s. The rotational speed for the contralateral group was doubled (ω_200%_ = 120°/s). For this group, a beep sound was created and played every 1.5 s (corresponding to a 180° turn) to ensure the surgeon kept up with the higher speed. If the surgeon felt severe resistance caused by the presence of a large amount of cortex or accumulation of debris (bone chips), the speed was decreased or the instruments were withdrawn, cleaned, and reinserted. This strategy followed the surgical guidelines and was applied in both groups.

### 2.4. Experimental Setup

**Figure 2:**
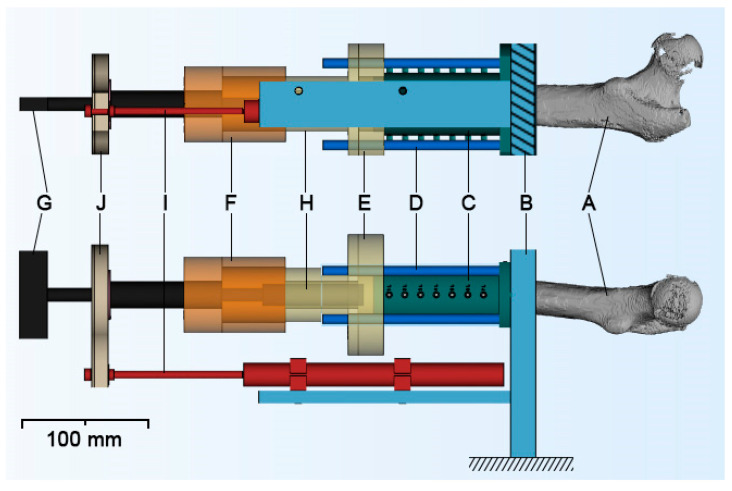
The femoral specimen (A) is mounted into the mounting platform (B) with the aid of drill guides (C) and four steel 8 mm rods (D). The drill guides accommodate one K-wire and six thermocouples each. Two flanges, one (E) attached to the drill guide and the steel rods and the other one (F) attached to the T-handle Hudson (G), allow exact alignment of the drills, the thread tap, and finally the implant (H). A displacement transducer (I) records the penetration depth of the instrument and the implant. It is firmly attached to the mounting platform, and its stroke is connected with the T-handle through a bearing (J).

The experimental setup is illustrated in Figure 2.

**Figure 3:**
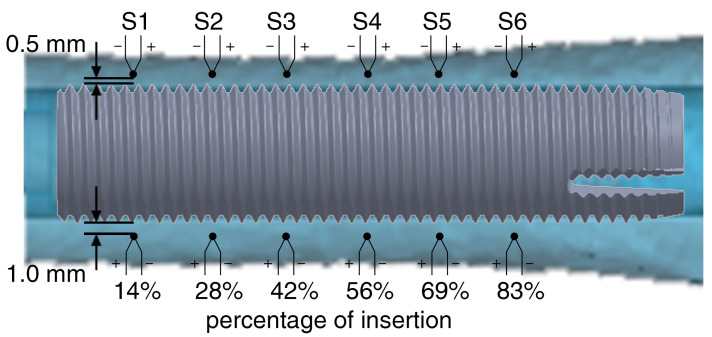
Position and labeling of 12 thermocouples within the bone cortex and at different insertion depths in respect to the fixture’s final position. S1 represents the most distal and S6 the most proximal thermocouple.

Up to 12 0.075 mm K fine gauge thermocouples (RS Components, Sorby, UK) were used to monitor the absolute temperature in real-time. Drill holes for the thermocouples were created with the aid of drill guides printed with a UV-curing translucent material (RGD720) by a Polyjet 3D-printer Connex 3 Objet 500 (Stratasys Ltd., Eden Prairie, MN, USA) for medical devices. The drill guides were created based on the individual geometry of each specimen, allowing the thermocouples to be positioned at the same distance (up to six sensors at 0.5 mm and up to six sensors at 1.0 mm) from the fixture’s surface in its final position (Figure 3).

**Figure 4:**
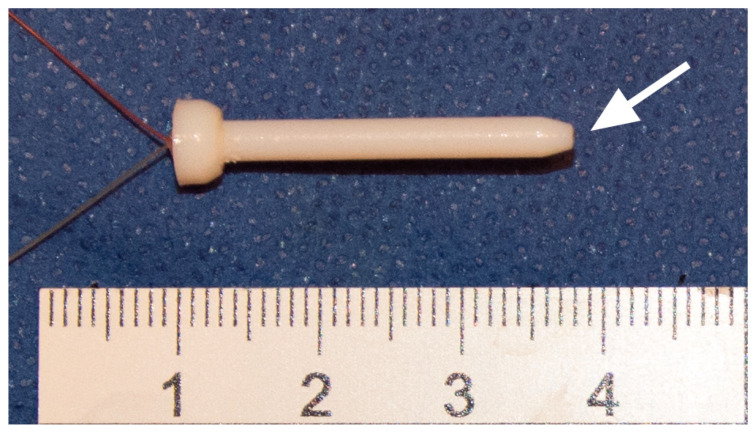
3D-printed sleeves of VeroWhite with incased thermocouple. The arrow indicates the exposed measuring point of the thermocouple.

Furthermore, the drill guides were firmly attached to the bone using two 3.0 mm K-wires to lock the specimen from any rotational or translational movement during the surgery. Finally, two cylindrical flanges, one attached to the drill guides and the other attached to the T-handle, were telescoped to align first the instruments and later the implant in its previously defined position. This ensured that the sensors were not damaged during bone preparation and the temperature was measured along the axis of the instruments or the implant at a constant distance. The thermocouples were incased into printed sleeves (2.5 mm in diameter) to facilitate their application and protect them from mechanical damage (Figure 4).

The measuring point of each thermocouple was exposed and in direct contact with bone tissue. A rectilinear displacement transducer PZ67-S (Gefran spa, Provaglio d'Iseo, Italy) was attached to the mounting platform. The stroke of the transducer was connected to the T-handle through a bearing and recorded the position of each instrument or the implant in the drill channel. Both the temperature and the displacement were digitalized with a sample rate of 10 Hz. Experiments were performed at room temperature (21.8 ± 1.0 °C). The temperature was recorded continuously, and the maximum temperature increase for each sensor was used in the data analysis.

### 2.5. Statistical Analysis

Differences in age, BMD, cortical thickness, and temperatures between the two groups and between the insertion depths were investigated with paired-samples t-test and the Wilcoxon signed ranks test for normally (ND) and non-normally distributed data (NND), respectively. Pearson product-moment correlation coefficient (R) was computed to investigate linear correlations between (1) age and maximum temperature increase, (2) BMD and maximum temperature increase, and (3) cortical width and maximum temperature increase for each specific sensor. Statistical significance was set at the 95% confidence level (95% CI), with *p*-values of <0.05. Each group was tested for normal distribution using the Shapiro-Wilk test. The analysis was performed using IBM SPSS Statistics 26 (IBM Corp., Armonk, NY, USA). Values given in the text are median, interquartile range (IQR = Q3–Q1), and range (min–max) (NND) or mean, standard deviation (SD), and range (min–max) (ND).

## 3. Results

Three out of twenty-four specimens were excluded. One specimen was too large and one was too small for the 18.0 mm implant. This resulted in very low and very high resistance in the last surgical steps and correspondingly low and high temperature peaks, respectively. One specimen rotated within the setup, causing damage to the sleeves and the thermocouples inserted therein.

There were no statistically significant differences in BMD, or cortical width (Ct.Wd.) between the two groups (see Table 1).

**Figure 5:**
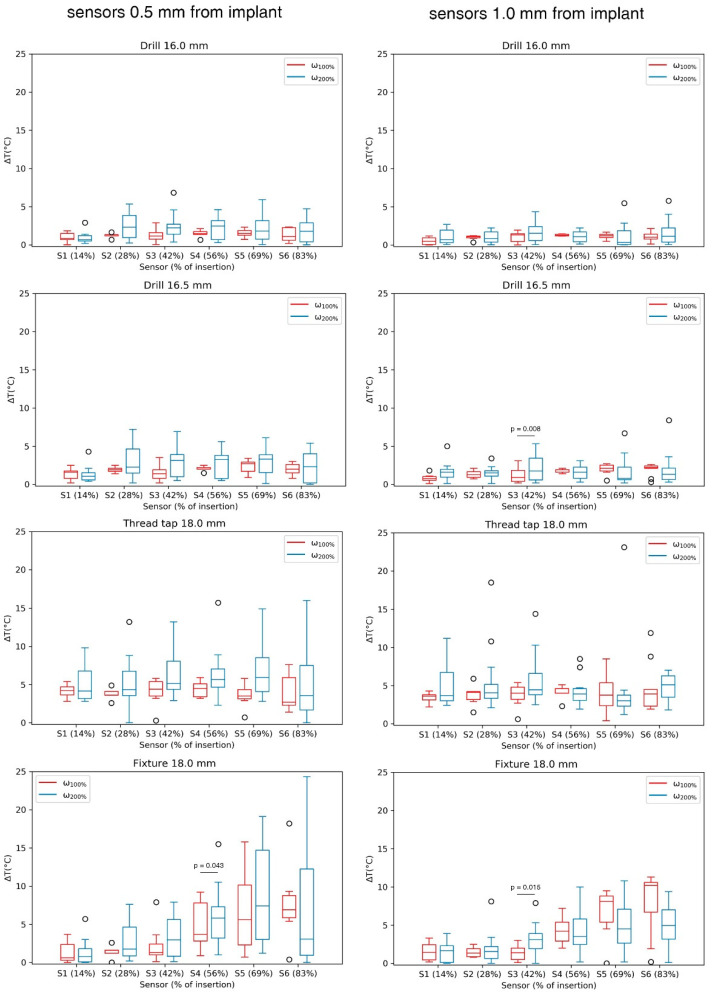
Maximum temperature increase measured for the final four surgical steps measured by 12 thermocouples (6 at 0.5 mm and 6 at 1.0 mm from the fixture's surface). Each box denotes the 25th through 75th percentiles, the bar within the box denotes the median value, and the whiskers extend 1.5 times the interquartile range above and below the 75th and 25th percentiles. Observations beyond the whiskers are plotted individually.

Generally, each consecutive surgical step led to an increase in temperature, which, however, remained below 5 °C. The difference in temperature increase between the two speed groups for each sensor was not significant in most cases for the sensors placed at 0.5 mm (0.063 < *p* < 1.000) and 1.0 mm (0.066 < *p* < 1.000) from the implant surface. The significant differences are marked in Figure 5. An increase in speed led to higher temperature values during 13.5, 16.0 and 16.5 mm drilling and during thread tapping (Table 2).

**Figure 6:**
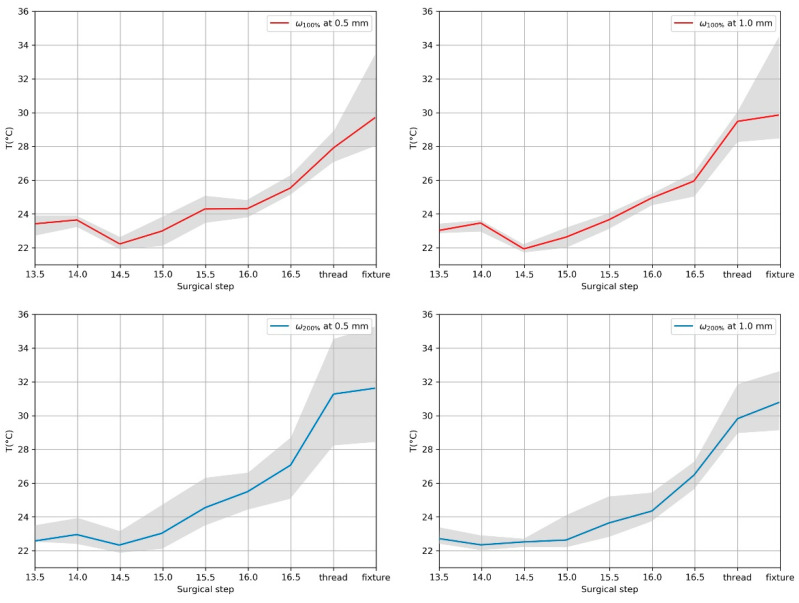
Cumulative heat generation during the simulated surgery for the two different speeds at 0.5 and 1.0 mm from the implant surface. The solid lines represent the median, and the shaded area represents the corresponding 25th to 75th percentile.

Figure 6 shows the absolute temperature increase during the installation procedure starting from room temperature. The cumulative temperature increase for all drilling steps reaches median temperatures <4 °C. The highest increase in cumulative temperature occurs during thread tapping.

For the correlation analysis, data were grouped in such a way that to each group the temperature increase, measured by one specific sensor (i.e., S1 to S6), at a specific distance from the fixture (0.5 and 1.0 mm), at a specific applied speed (ω_100%_ and ω_200%_), as well as the specific surgical step (13.5 mm drilling to fixture insertion), was assigned.

In consequence, each group was correlated with BMD, age, and cortical width. Applying the aforementioned variables resulted in 216 combinations, which were tested in the correlation analysis. The majority of the tested combinations did not show statistically significant correlations. However, BMD was predictive of temperature increase in 19 cases (8.8%) (0.33 < R² < 0.77, 0.003 < *p* < 0.049). Cortical width correlated with the temperature increase in 14 cases (6.5%) (0.55 < R² < 0.87, 0.002 < *p* < 0.047) and donors’ age in 8 cases (3.7%) (0.38 < R² < 0.89, 0.004 < *p* < 0.042), whereas the age was negatively correlated with the maximum temperature increase.

## 4. Discussion

During the surgical procedure for the installation of the fixture of a bone-anchored prosthesis, surgeons are trained to maintain a very low speed during drilling, thread tapping, and fixture insertion aiming to minimize the amount of heat generated within the bone tissue. Heat-induced tissue injuries endager primary healing and osseointegration, and consequently the implant stability. Presented data quantify for the first time the temperature increase for each surgical step at the clinically practiced speed and when that speed is doubled.

There is no general consent on the temperature threshold for heat-induced injuries in bone, and the literature on the subject is outdated. Moreover, the mechanisms underlying the tissue effects of thermal necrosis are not completely understood. The main cellular factors are considered to be changes in protoplasmic proteins, with inactivation of enzymes and metabolic processes, and alterations in protoplasmic lipids, occurring from water being vaporized [6]. Denaturation of different bone proteins is reported to occur between 40 and 60 °C [7,8,9]. Lundskog [10] studied the diaphorase activity of bone tissue subjected to point thermal insults at various exposure times and temperatures. Exposing bone to 80 °C between 5 and 30 s had a negative effect on the diaphorase activity of osteocytes, whereas the radius of deactivation increased with exposure time. Similarly, in the same study, bone was exposed to heat with a constant exposure time of 30 s. Diaphorase deactivation was initiated at 50 °C. Cortical necrosis and necrosis of osteocytes were reported in living rabbit bone if heated to 47 and 55 °C for 5 and 1 min, respectively [5]. A review of the literature concluded that most authors consider 50 °C to be the temperature at which bone necrosis occurs [6]. Thus, an increase of around 13 °C above body temperature would initiate bone damage.

In the present study, a temperature increase was observed between each two consecutive surgical steps. The temperature increase during drilling was ascending with each consecutive drill size but stayed well below 2.5 °C and a threshold to possibly jeopardize bone healing. The ascending temperature increase between each two consecutive drilling steps is the consequence of the increased amount of cortical bone that is being removed [11]. In the presented sample, an average of 1.0 °C (at ω_100%_) and a maximum of twelve different drill sizes were used to reach the final canal size. Thus, drilling represents by far the most time-consuming part of the surgery. Considering the fact that doubling the clinically recommended drilling speed does not lead to an increase in temperature and that the cumulative temperature stress during drilling remains limited, it can be concluded that drilling speed can be increased to shorten the intervention time. However, these results must not be read as one-directional; rather, it must be assumed that higher speeds will generate more heat, although this was not the case at the presented magnitude of rotational speed.

Significantly higher temperatures were observed during thread tapping and fixture insertion. The median increase in temperature remained below 5 °C in these two groups but revealed local temperature maxima exceeding 10 °C above initial (room) temperature. Thus, despite a low median temperature increase, it is not recommended to increase the speed during these two steps with the highest risk potential for overheating, due to the aforementioned temperature peaks. Furthermore, the cumulative temperature increase is significant after thread tapping. Moreover, a significant temperature increase was assessed for the higher speed during thread tapping. Tapping the thread for the used 18 × 80 mm BioHelix fixture with a 1.75 mm pitch requires around 57 full rotations generating a great amount of frictional force. The heat that is generated at the bone-instrument interface is not being removed by bone chips or a cooling liquid but is completely conducted into the bone tissue. Thus, the temperature measured at different distances to the implant is nearly identical, and a thermal injury would not only occur at the implant interface but also in deeper layers of the bone. During this step, a frequent withdrawal of the instruments will allow the heat to escape the instrument and, more importantly, the bone chips, which store the most amount of heat during thread tapping and also increase the friction forces and shearing energy [11], to be removed from the instrument. Additionally, the use of cooled instruments could be considered for the surgical steps with the highest accumulation of heat.

During the experiments, it was observed that the highest temperature was generated at the tip of the instrument and was being transferred from distal to proximal or from the sensor S1 to the sensor S6. This observation is not clearly visible for most surgical steps as the instruments were frequently withdrawn to remove the bone chips, allowing the bone and the instrument to cool down and thus preventing temperature peaks. Fixture insertion is the only step where no withdrawal occurs, and an increase in median temperature from S1 to S6 is obvious. From this perspective, the proximal part of the femur is more likely to suffer thermal injuries. This again underlines the necessity to frequently withdraw and clean the instruments, as advised by the manufacturer.

Defining a patient-specific variable that correlates with the generated heat in the bone tissue would allow for preoperative speed selection and a more accurate prediction of surgical duration. Therefore, BMD, age, and cortical width of the donors were correlated with the locally measured temperature increase. There was only a limited ability to predict patient-specific temperature increase using these quantities as predictor variables. BMD showed a positive statistically significant correlation with peak temperatures in about 10% of the cases. It seems plausible that the volume of removed bone including its local density would allow for higher precision in temperature prediction than local bone width and its global density. Literature on this particular topic is scarce. Tests on artificial bones using small drill sizes (2.5–4.5 mm) have shown that higher temperatures are generated with higher material density [12]. The authors assume that besides bone quality, reflected by BMD, patient’s age, and bone width, the information on bone curvature and the resulting exact amount of removed bone tissue during the specific step is necessary for a more accurate prediction. Even though technically feasible, collecting these data in preoperative planning would not be feasible in everyday clinical practice.

This study has several limitations. The first limitation arises from the study type. An in vitro study is affected by the high age of the donors and corresponding bone quality that does not reflect the typical patient who would receive a bone-anchored prosthesis. Moreover, it was not possible to simulate the thermal effect of blood flow within the bone or the thermal conductivity of the surrounding soft tissue. However, heat transfer, especially due to frequent branching and joining of vascular networks in the diaphysis maintaining low blood velocity, is likely negligible [13,14]. Second, the position of the sensors was calculated based on QCT images at 0.6 mm slice thickness. This currently represents the highest resolution that can be used clinically and could have resulted in inaccuracies in positioning of the thermocouples. Third, variables such as amputation height, duration of each surgical step, and number of instrument withdrawals could not be standardized. The amputation height is based on the primary amputation and the patient’s bone curvature and cortical thickness. Clinically, the aim is to position the fixture in the bone and ensure a minimum of 2 mm remaining cortical thickness. The number of instrument withdrawals is clinically not standardized either, as they are performed whenever high resistance is felt by the surgeon. The instrument is then cleaned in order to prevent bone from bursting. Nevertheless, the aforementioned variables represent confounding variables that might have had an effect on the generated heat.

## 5. Conclusions

In conclusion, by measuring the amount of heat generated during the preparation for and insertion of osseointegrated transfemoral implants, it was shown that the clinically recommended speed generates rather low temperature peaks. The data suggest that the drilling speed could be further increased without endangering bone tissue quality through thermal necrosis. This could significantly shorten the duration of the surgery. Finally, frequent withdrawing of the instrument to remove the bone debris prevents temperature peaks from arising.

## Figures and Tables

**Table 1:** Description of the specimens included in the two groups: ω_100%_ and ω_200%_. A statistically significant difference between the median or mean of the two groups was not observed (*p* > 0.05). Values given in the text are median, interquartile range (IQR = Q3–Q1), and range (min–max) (NND) or mean, standard deviation (SD), and range (min–max) (ND).

	ω_100%_	ω_200%_	Stat. Sign.
n	10	11	-
Age (years)	81.2 ± 12.6 (65–101)	80.3 ± 12.2 (65–101)	-
BMD (g/cm^2^)	0.71 (IQR: 0.59–0.78) (0.49–0.94)	0.75 (IQR: 0.65–0.81) (0.65–0.90)	0.155
Ct.Wd. (mm)	7.6 ± 1.2 (6.0–9.9)	8.1 ± 1.2 (5.9–9.7)	0.059 (−0.92–0.02)

**Table 2:** The median for the maximum temperature increase in °C for each surgical step at two different speeds, the inter-quartile range (IQR), and the range (min–max). Statistically significant differences were found for the 13.5, 16.0, and 16.5 mm drills and thread tap.

	Sensor at 0.5 mm from Implant			Sensor at 1.0 mm from Implant		
	ω_100%_	ω_200%_	Stat. Sign.	ω_100%_	ω_200%_	Stat. Sign.
Drill 13.5 mm (n = 13)	0.5 (IQR: 0.1–1.0) (0.2–1.8)	0.3 (IQR: 0.2–1.0) (0.0–4.5)	0.527	0.3 (IQR: 0.1–0.6) (0.0–1.5)	0.5 (IQR: 0.2–1.2) (0.0–3.4)	0.002 *
Drill 14.0 mm (n = 16)	0.5 (IQR: 0.1–0.8) (0.0–2.8)	0.6 (IQR: 0.2–1.6) (0.00–4.3)	0.339	0.5 (IQR: 0.0–0.7) (0.0–1.6)	0.3 (IQR: 0.0–0.9) (0.0–2.9)	0.083
Drill 14.5 mm (n = 20)	0.5 (IQR: 0.2–0.9) (0.0–2.0)	0.3 (IQR: 0.0–1.1) (0.0–4.4)	0.845	0.4 (IQR: 0.2–0.7) (0.0–1.7)	0.3 (IQR: 0.0–0.5) (0.0–3.2)	0.121
Drill 15.0 mm (n = 21)	1.2 (IQR: 0.3–2.0) (0.0–4.8)	0.7 (IQR: 0.1–2.4) (0.0–7.0)	0.247	0.9 (IQR: 0.3–1.5) (0.0–3.8)	0.4 (IQR: 0.0–1.9) (0.0–5.5)	0.196
Drill 15.5 mm (n = 21)	1.5 (IQR: 0.8–2.3) (0.1–5.4)	1.6 (IQR: 0.2–3.4) (0.0–5.6)	0.103	1.1 (IQR: 0.6–1.5) (0.1–4.2)	0.9 (IQR: 0.1–2.5) (0.0–5.5)	0.335
Drill 16.0 mm (n = 21)	1.2 (IQR: 0.7–1.7) (0.0–2.8)	1.6 (IQR: 0.5–2.8) (0.0–6.8)	0.010 *	1.0 (IQR: 0.6–1.3) (0.0–2.1)	0.8 (IQR: 0.2–1.9) (0.0–5.7)	0.118
Drill 16.5 mm (n = 21)	1.7 (IQR: 1.3–2.5) (0.1–3.5)	2.2 (IQR: 0.7–3.8) (0.0–7.2)	0.008 *	1.6 (IQR: 0.7–2.1) (0.1–3.1)	1.4 (IQR: 0.6–2.2) (0.1–8.4)	0.044 *
Thread tap 18.0 mm (n = 21)	3.9 (IQR: 3.1–5.0) (0.2–7.6)	4.9 (IQR: 3.4–8.2) (0.0–16.0)	0.006 *	3.9 (IQR: 2.7–4.5) (0.4–11.8)	3.8 (IQR: 3.0–5.9) (1.1–32.2)	0.012 *
Fixture 18.0 mm (n = 21)	2.6 (IQR: 0.9–6.4) (0.0–18.1)	3.1 (IQR: 1.1–6.8) (0.0–24.3)	0.123	2.3 (IQR: 0.9–7.0) (0.0–11.3)	2.9 (IQR: 1.3–4.8) (0.0–10.8)	0.835

## Data Availability

The data presented in this study are available on request from the corresponding author. The data are not publicly available due to national privacy regulations.
